# In Vitro Antimicrobial Photodynamic Therapy for *Pseudomonas aeruginosa* (*P. aeruginosa*) and *methicillin-resistant Staphylococcus aureus* (MRSA) Inhibition Using a Green Light Source

**DOI:** 10.3390/pharmaceutics16040518

**Published:** 2024-04-09

**Authors:** Karen Roa-Tort, Yael Saavedra, Angélica Villanueva-Martínez, Adriana Ganem-Rondero, Laura Abril Pérez-Carranza, José M. de la Rosa-Vázquez, Gabriel Ugalde-Femat, Omar Molina-Alejandre, Andrea Angela Becerril-Osnaya, Josué D. Rivera-Fernández

**Affiliations:** 1 Laboratorio de Optomecatrónica, UPIIH, Instituto Politécnico Nacional, Distrito de Educación, Salud, Ciencia, Tecnología e Innovación, San Agustín Tlaxiaca 42162, Mexico; kroat@ipn.mx; 2 Laboratorio de Biofotónica, ESIME ZAC, Instituto Politécnico Nacional, Gustavo A. Madero, Ciudad de México 07738, Mexico; rgarcias1401@alumno.ipn.mx (Y.S.); josdelarosa@yahoo.com (J.M.d.l.R.-V.); gugaldef1300@alumno.ipn.mx (G.U.-F.); 3 Laboratorio de Investigación y Posgrado en Tecnología Farmacéutica (L-322), Facultad de Estudios Superiores Cuautitlán, Universidad Nacional Autónoma de México, Cuautitlán Izcalli 54740, Mexico; angelynca1024@gmail.com (A.V.-M.); ganemq@hotmail.com (A.G.-R.); omar.alej@hotmail.com (O.M.-A.); 4 Laboratorio de Bacteriología Diagnóstica de la Sección de Ciencias de la Salud Humana (anexo al L-513, Campo1), Facultad de Estudios Superiores Cuautitlán, Universidad Nacional Autónoma de México, Cuautitlán Izcalli 54740, Mexico; lapc.bqd@gmail.com

**Keywords:** in vitro antimicrobial photodynamic therapy, bacterial inhibition, *P. aeruginosa*, *methicillin-resistant Staphylococcus aureus* (MRSA), green light aPDT

## Abstract

Photodynamic therapy (PDT) has been based on using photosensitizers (PS) and applying light of a specific wavelength. When this technique is used for treating infections, it is known as antimicrobial photodynamic therapy (aPDT). Currently, the use of lighting sources for in vitro studies using aPDT is generally applied in multiwell cell culture plates; however, depending on the lighting arrangement, there are usually errors in the application of the technique because the light from a well can affect the neighboring wells or it may be that not all the wells are used in the same experiment. In addition, one must be awarded high irradiance values, which can cause unwanted photothermal problems in the studies. Thus, this manuscript presents an in vitro antimicrobial photodynamic therapy for a *Pseudomonas aeruginosa* (*P. aeruginosa*) and *methicillin-resistant Staphylococcus aureus* (MRSA) inhibition study using an arrangement of thermally isolated and independently illuminated green light source systems for eight tubes in vitro aPDT, determining the effect of the following factors: (i) irradiance level, (ii) exposure time, and (iii) Rose Bengal (RB) concentration (used as a PS), registering the *Pseudomonas aeruginosa* (*P. aeruginosa*) and *methicillin-resistant Staphylococcus aureus* (MRSA) inhibition rates. The results show that in the dark, RB had a poor antimicrobial rate for *P. aeruginosa*, finding the maximum inhibition (2.7%) at 30 min with an RB concentration of 3 µg/mL. However, by applying light in a correct dosage (time × irradiance) and the adequate RB concentration, the inhibition rate increased by over 37%. In the case of MRSA, there was no significant inhibition with RB in complete darkness and, in contrast, the rate was 100% for those experiments that were irradiated.

## 1. Introduction

The use of technology in the medical field has evolved, intending to find more effective methods for the study of therapies for various pathologies. An example is the implementation of photonic instrumentation in cell cultures under photodynamic therapy (PDT) [1].

PDT is a procedure that is clinically used in the treatment of several oncological human diseases, such as skin, esophageal, head and neck, lung, and bladder cancers [2]. Moreover, PDT has several non-oncology applications [3], including the treatment of non-cancerous human diseases, such as dermatologic disorders (e.g., acne [4], warts [5], photoaging [6], psoriasis [7], vascular malformations [8], hirsutism [9], keloid [10], and alopecia areata [11]), ophthalmic problems (central serous chorioretinopathy [12] and corneal neovascularization [13]), and cardiovascular conditions (atherosclerosis [14] and esophageal varix [15]). This type of therapy dates back over 3000 years ago, because Egyptians, Indians, and Chinese used it for skin condition treatments [16,17].

PDT is based on the use of photosensitizers (PSs), which are molecules that have electrons in their structure that, when subjected to light of a specific wavelength, pass to an excited singlet state and then undergo a crossover between systems, reaching a triplet state of lower energy, but longer lasting. Subsequently, the triplet state undergoes a type I photochemical reaction, producing superoxide radicals ($O_{2}^{-}$), or type II, producing singlet oxygens (^1^$O_{2}$) [18]. In either case, the generated reactive oxygen species (ROS) can be used to cause apoptosis or necrosis of certain cells, e.g., tumor cells, bacteria, fungi, and parasites, thus representing an interesting approach not only for the treatment of infections but also for various pathologies [19]. When this technique is used for the treatment of infections, it is known as antimicrobial photodynamic therapy (aPDT).

Among several PSs, RB is an anionic dye derived from fluorescein, with an absorption/emission maximum in water at 540/567 nm. It has several advantages such as being inexpensive, biocompatible, and widely available, in addition to its ability to inhibit both resistant Gram-positive and Gram-negative strains [20].

The PS and the light source are important for PDT. The selection of both the PS and the wavelength of the light source depends on the type of treatment or study being performed, e.g., if the study is in vivo, in situ, in vitro, etc., making the light source configuration an essential factor that will determine the quality of the obtained results [21].

In vitro aPDT studies usually use cell culture plates and test tubes, in which samples are usually placed on plates and the light source is located under the plate, but due to the configuration of these, contiguous wells are not usually used to prevent light from affecting neighboring wells or the lighting source is changed to be able to irradiate the plate from all directions, thus requiring a more powerful lighting source [22].

The above implies that clinical efficacy is highly dependent on the accuracy of the irradiation dose, which translates into light fluence (J/cm^2^), irradiance (W/cm^2^), exposure time (s), wavelength (nm), light source (Laser, lamps, LED, OLED, daylight, etc.), illuminated area (cm^2^), and distance between the light source and the sample (cm or mm).

A characteristic that has great importance in the lighting source is the irradiance, which is the incident energy per second per unit area expressed in Watts per cm^2^, as various studies have indicated that low irradiance benefits the implementation of PDT [23,24,25,26,27].

The main reason for low efficiency in the implementation of PDT is the use of high irradiance since it causes oxygen depletion in the tissues, leading to undesired photodegradation of the PS. Furthermore, this same condition increases the possibility of affecting the sample by having a direct impact on the dominant mechanism of cell death during PDT [28].

**Figure 1:**
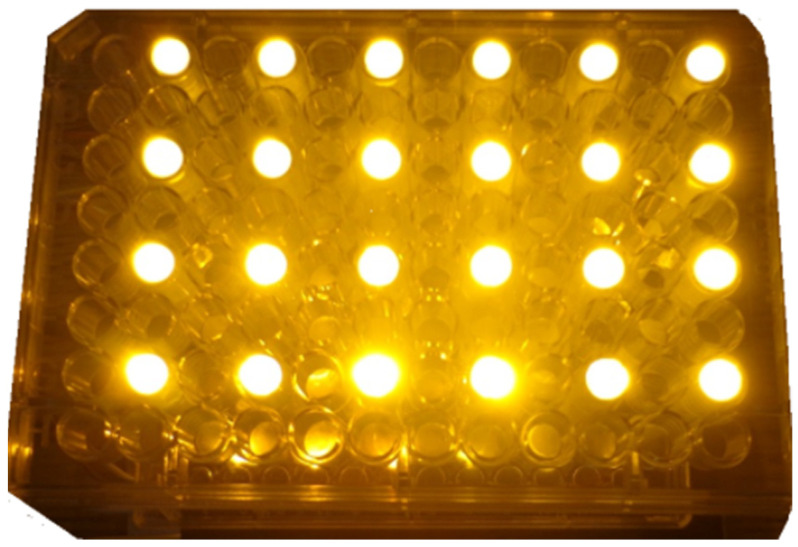
Example of cell culture plate irradiated with underutilization of available wells.

PDT can be performed with various types of light sources, including the use of lasers, incandescent lighting sources, and light-emitting diodes (LEDs) [27]. Using laser light can be expensive and requires optical systems to expand the light beam to irradiate a larger area. On the other hand, incandescent lighting sources can cause undesirable thermal effects on the PDT. For this reason, a better option is usually the use of LED lighting sources, as they are less expensive, cause less of a thermal effect, and allow easy adaptation in matrix configurations for cell culture dishes [29,30,31]. Some lab companies have developed light sources with this configuration [32]. Occasionally, researchers fabricate their own illumination sources for the irradiation of cell culture wells; however, they do not consistently utilize all wells, as the emitted light from one may interfere with neighboring elements. In some cases, it is necessary not to irradiate continuous wells. An example of this situation can be seen in Figure 1.

As Figure 1 shows, the light is irradiated over the entire cell culture plate, which causes light from one well of the plate to interfere with the next, which means that if one wants to perform different tests in each well, it would be impossible to use all wells; this will reduce the number of experiments per well plate, causing a greater investment in the number of inputs per experiment. An alternative would be to concentrate the light on each well by placing elements that block light between wells; notwithstanding, increasing the fluence rate of light could be bad for PDT.

On the other hand, in vitro experiments could be run using well plates, test tubes, and even vials, depending on how the experiment was set up. This has led to the development of lighting sources for PDT that allow direct irradiation of samples in test tubes or in vials [32].

It is perceptible that the light source in Figure 1 irradiates the whole system, so the light for two continuous vials can affect the experiment of each of them. In addition, the vials are usually used for low capacities, unlike a test tube. In addition, these lighting sources usually have a fixed or uncontrolled irradiation intensity to turn on a specific LED for the tube or vial of interest.

For this reason, this manuscript focuses on the design, characterization, and implementation of a green light source for in vitro aPDT in test tubes, with the purpose of evaluating the effect of the following factors: (i) irradiance level, (ii) exposure time, and (iii) RB concentration, registering the percentage of microbial inhibition of two resistant bacteria, *Pseudomonas aeruginosa* (*P. aeruginosa*) and *methicillin-resistant Staphylococcus aureus* (MRSA).

It is essential to note that another motivation behind this investigation stems from the high costs associated with commercial systems, which often lack the flexibility to accommodate in vitro processes using test tubes. Therefore, the development of our system presents a significant advantage in terms of cost-effectiveness, offering a low-cost solution for conducting aPDT treatments with precise control of the light source parameters.

## 2. Materials and Methods

### 2.1. In Vitro Antimicrobial Photodynamic Therapy

#### 2.1.1. Photosensitizer (PS)

RB disodium salt was used as the PS for this study. For this, a stock solution of RB was prepared in distilled water, from this preparing three diluted solutions (6.0, 4.76, and 3.5 μg/mL) in a sterile physiological saline solution for the aPDT study.

#### 2.1.2. Pathogens Cell Culture

The antimicrobial effect of RB with PDT was tested on two bacterial strains: *P. aeruginosa* and MRSA. The strains were provided by the Bacteriological Diagnostic Service for the internal and external population of the Facultad de Estudios Superiores Cuautitlán (National Autonomous University of México, UNAM). Both infectious agents were cultured on Muller Hinton agar by the streak plate method, incubating the plates at 37 °C for 24 h.

#### 2.1.3. Protocol for aPDT

With the purpose of evaluating the effectiveness of the equipment for PDT, the effect of (i) the irradiance level, (ii) the exposure time, and (iii) the concentration of RB, the microbial inhibition was determined. For this, a factorial experimental design was proposed (one per pathogen) using Statgraphics^®^ Centurion XVI software, version 16.1.03, as shown in Table 1. All experiments were performed in duplicate.

#### 2.1.4. Bacterial Photosensitization and Viable Cell Counting

The bacterial suspension was prepared using a young bacterial culture, adjusting the turbidity to 0.5 with physiological saline using a McFarland nephelometer. Then, the bacterial suspension was mixed in a sterile tube with the PS solution in a 1:1 (*v*/*v*) ratio. This mixture was treated in the aPDT conditions shown in Table 1, according to the proposed experimental design.

After PS of the mixture, decimal dilution series were carried out for MRSA (10^−3^, 10^−4,^ and 10^−5^) and *P. aeruginosa* (10^−4^, 10^−5^, and 10^−6^), which were then inoculated onto standard methods agar plates according to the spread plate protocol. Once the plates were inoculated, they were incubated at 37 °C for 24 h, counting plates that contained between 30 and 300 bacterial colonies. The counts were expressed as colony-forming units (CFU), reporting results in CFU/mL.

### 2.2. Green Light Source Characterization

#### 2.2.1. Wavelength

In the case of wavelength, the Ocean Insight^®^ brand QE65000 spectrometer (Dunedin, FL, USA) was used; both the control of the instrument and the capture of the data from the excitation source were carried out using OceanView ^®^ software, version 1.6.7.

**Figure 2:**
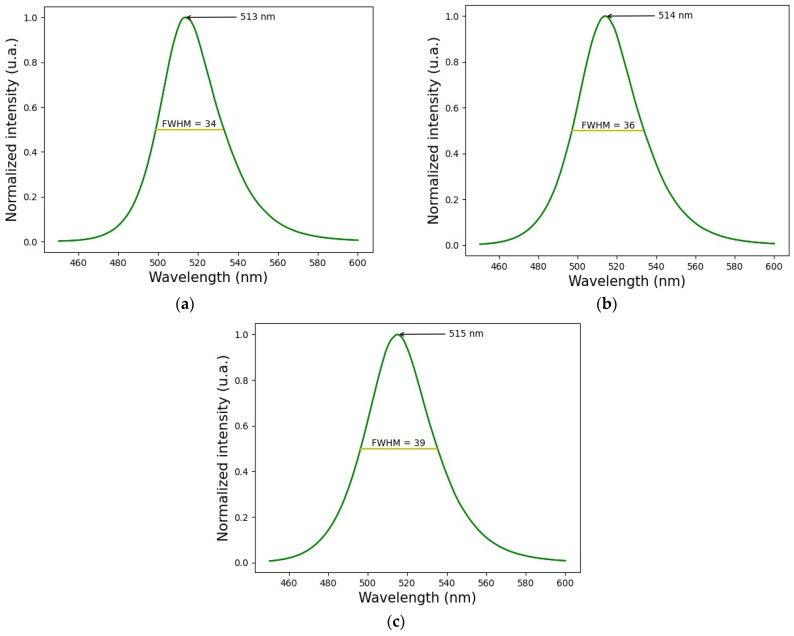
Wavelength characterization, (**a**) at 1 W, (**b**) at 2 W, and (**c**) at 3 W of electrical power.

The measured wavelength was 514 ± 1 nm, with a Full Width Half Maximum (FWHM) of 36 ± 3 nm. The variation obtained both in the displacement of the wavelength and in the FWHM is derived from the fact that measurements were made with 1 W, 2 W, and 3 W of electrical power of the LED. Figure 2 shows the (a) measurement of wavelength at 1 W, (b) 2 W, and (c) 3 W.

#### 2.2.2. Light Source Stability

**Figure 3:**
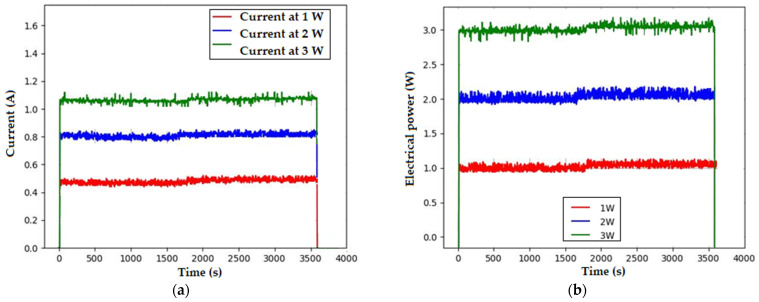
Electrical stability of the system during 1 h of operation time, (**a**) current stability, and (**b**) electrical power stability.

Measurements for current and electrical power were developed to verify the stability during the operating time, and it is important to note that every test was conducted for 1 h of continuous operation. Figure 3 shows these (a) current and (b) electrical power values. It is noticeable that variations exist, but they are negligible for the purposes of the system; the standard deviation for current has a maximum value of ±0.07 A, while electrical power has ±0.1 W.

#### 2.2.3. Light Source Principal Parameters

The light source in this work for in vitro aPDT processes is not limited solely to this type of study since it allows for a variation in the amount of irradiance delivered. The selected light-emitting diodes were from the manufacturer SiLED model LED-P3G200-120/41 whose wavelength is in the range of 490 to 590 nm (with an emission peak at 514 nm). They are high-power LEDs that allow the consumption of electrical power of up to 3 W. This value is adjustable and variable according to the electrical configuration selected for its control, allowing the irradiance of the LED to be varied and therefore the fluence. Table 2 shows the main characteristics of the green light source used in this work.

**Figure 4:**
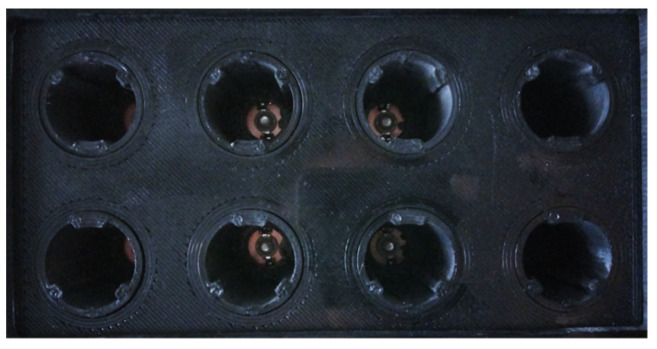
LED array for eight test tubes using a 3D printing lab rack grid.

The green light source for in vitro aPDT has a capacity of eight test tubes. However, the design of the source is not limited to this number and each tube has one LED, and the set of these forms an arrangement of two rows by four columns, as shown in Figure 4. It is possible to observe that the LED array for the eight tubes has a 3D printing lab rack grid that allows each tube to be separated and supported, providing the characteristic of isolating the light of one tube and that of the other, avoiding unnecessary light problems during experimentation in a specific test tube.

#### 2.2.4. Light Source Control

**Figure 5:**
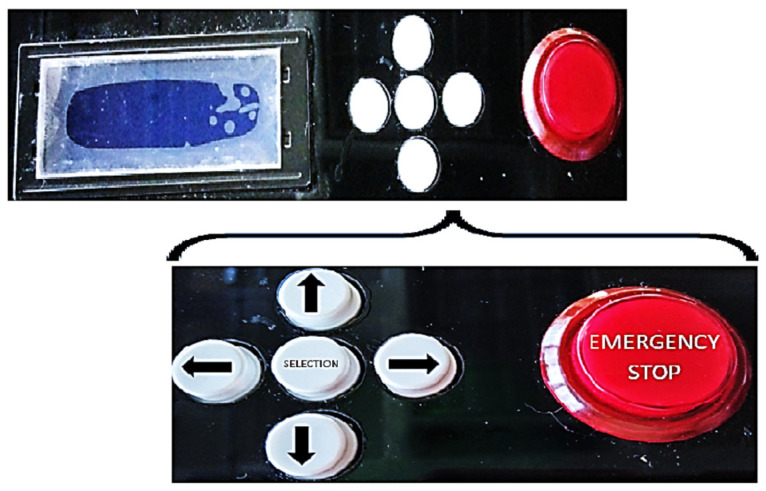
Control panel buttons and LCD screen.

Control for each LED was implemented using the microcontroller PIC16F15355. This was used because of the number of input and output ports both digital and analog and its capacity to program memory and RAM memory for the functions. Moreover, we decided to design the source as a portable system for simple hauling as well as easy operation for the user. An LCD screen was placed on the system, which allows for observing the configuration of the system parameters. These parameters are selectable using a panel of five buttons, as can be seen in Figure 5. The four external buttons allow scrolling through menus between the options that are displayed on the screen, while the central button allows for selecting the configuration menu and setting the parameter that has been selected. Moreover, an emergency stop button was added.

To select each LED and monitor its parameters, namely voltage, current, and temperature, CD4051 digital multiplexer circuits were used; likewise, the temperature was monitored by means of the LM35 sensor per tube. On the other hand, voltage and current values were monitored using analog input ports of the microcontroller. To control the irradiation power of each LED, a trans-impedance operational amplifier was used (control current fed back by a resistance detector), to which the required value is sent from the microcontroller, using a Digital-Analog Converter (DAC), to set the current and voltage values necessary to establish the irradiance level.

The LEDs selected for the system presented in this manuscript have a 514 ± 1 nm wavelength with a Full Width Half Maximum (FWHM) of 36 ± 3 nm, and this wavelength was selected because RB was used as the PS. On the other hand, the irradiance can be variable with values from 10 ± 0.5 mW/cm^2^ to 60 ± 0.5 mW/cm^2^.

**Figure 6:**
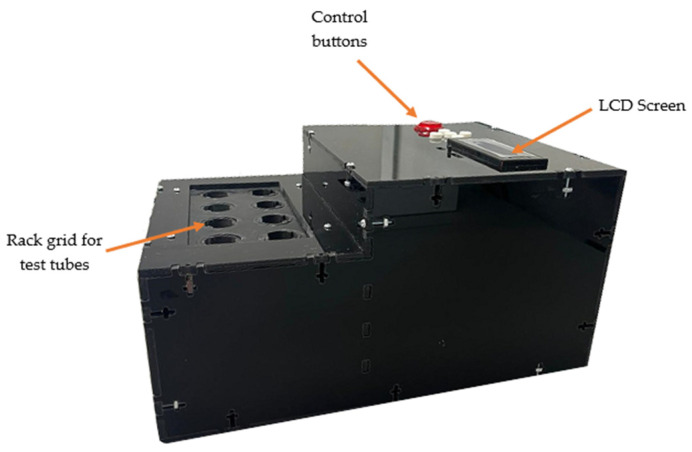
Green light source system for in vitro aPDT studies.

The irradiation light source is portable, of which the dimensions are 250 mm in width, 347 mm in length, and 180 mm in height. It can be connected to a 120 V alternating current (AC) power outlet and operates continuously for 1 h without modifications that affect its operation. It is possible to select exposure times in 2.5 min increments up to the maximum time to facilitate the experiments. The green light source for in vitro aPDT using test tubes is shown in Figure 6.

**Figure 7:**
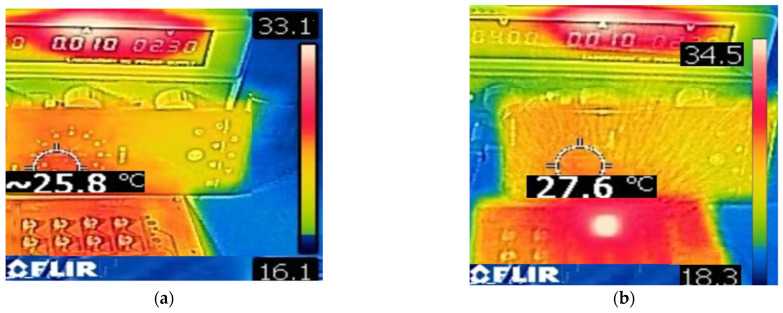
Comparison of temperature measurement when characterizing the light source, (**a**) system off, (**b**) system on at one hour of operation.

Temperature characterization was integral to the design and development phases of the system. These measurements were conducted before system operation and during a one-hour monitoring period with the LED activated, as illustrated in Figure 7. This monitoring revealed an increase in temperature of approximately 2 °C. Temperature measurements were captured using a FLIR^®^ (Wilsonville, OR, USA) model C3X infrared camera.

## 3. Results

According to the results available in Table 3, regardless of the factors that were varied, the microbial inhibition of MRSA is complete. Therefore, in this case, and considering economic and time issues, to eliminate MRSA, it would be preferable to use the minimum conditions, i.e., shorter time, lower irradiance, and lower RB concentration (Experiment D).

To evaluate the effect of the factors exposed in the methodological part, which are considered to affect the effectiveness of aPDT, tests outlined in the experimental design were carried out randomly, obtaining the results shown in Table 4 for *P. aeruginosa*.

**Figure 8:**
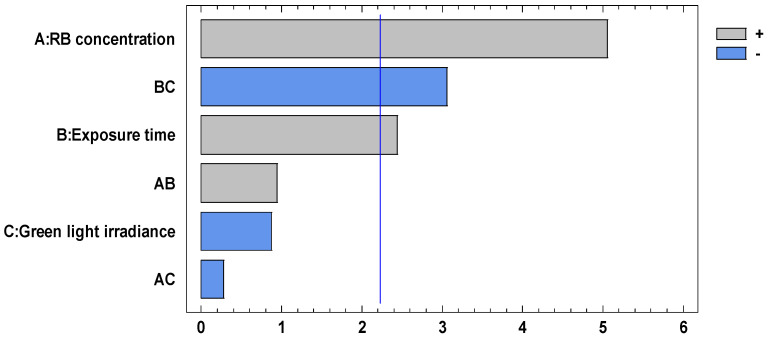
Pareto chart for *P. aeruginosa* microbial inhibition.

Table 5 shows the Analysis of Variance (ANOVA) performed using Statgraphics^®^ Centurion XVI software, version 16.1.03, showing the factors that present *p* values < 0.05 and therefore influence microbial inhibition: RB concentration (A), exposure time (B), and the interaction exposure time of green light irradiance (BC). This is clearly illustrated in the Pareto chart shown in Figure 8, where factors A, B, and BC exceed the limit value of t (blue line), with the RB concentration (factor A) having the greatest impact on microbial inhibition followed by the interaction BC and the exposure time (B). The interaction factor (BC) is better known as light fluence (J/cm^2^) and is an important parameter in establishing an effective aPDT.

These results show that the RB concentration and the time-irradiance interaction play a fundamental role in determining the optimal conditions that allow maximum microbial inhibition to be achieved. Both the time and irradiance can be controlled using the aPDT equipment, adjusting these values according to the desired results.

To find the best conditions to maximize the degree of microbial inhibition for *P. aeruginosa*, an equation depending on the factors previously found could be written. Equation (1) was a function of the regression coefficient values calculated by Statgraphics^®^ Centurion XVI.

*P. aeruginosa* microbial inhibition rate = −55.0884 + 7.69519 * RB concentration + 0.0259377 * Exposure time − 0.00107195 * Exposure time * Green light irradiance (1)

**Figure 9:**
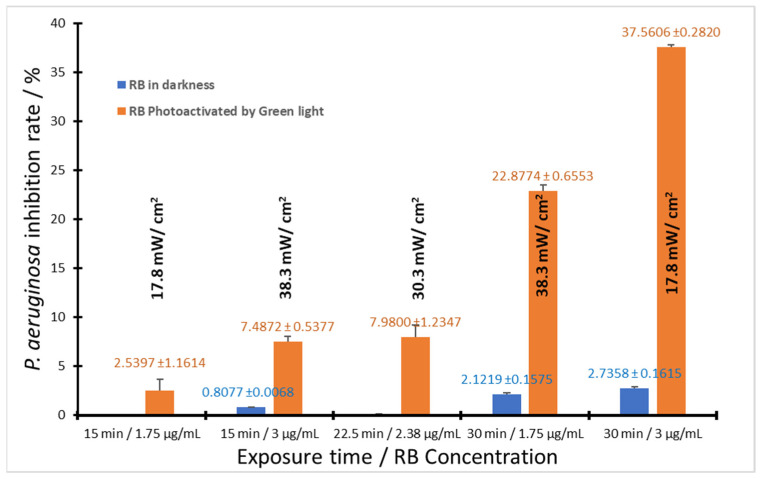
Comparison of the inhibition rate for *P. aeruginosa*, when treated with RB in darkness and RB photoactivated. Black letters show irradiance, orange letters show average *P. aeruginosa* inhibition ± standard deviation by RB Photoactivated, and blue letters show average *P. aeruginosa* inhibition ± standard deviation by RB in darkness, *n* = 2.

To compare the effect of light on the microbial inhibition of *P. aeruginosa*, a series of experiments were carried out, treating the bacteria with RB in complete darkness and RB activated by green light. The results are shown in Figure 9.

In the dark, RB has a very poor antimicrobial rate for *P. aeruginosa*, finding the maximum inhibition (2.7%) at 30 min with an RB concentration of 3 µg/mL. However, by applying light at the correct dosage (time × irradiance) and the adequate RB concentration, the inhibition rate increases dramatically by over 37%. In the case of MRSA, there was no significant inhibition in the assays with RB in complete darkness, and inhibition was 100% for those that were irradiated.

## 4. Discussion

Currently, it is common in PDT lighting sources for in vitro experiments to use LED technology because of its low cost and relative ease of implementation. Besides this peculiarity, a greater number of lighting sources are usually developed for the implementation of aPDT in multiwell cell culture plates [31,32,33,34,35] and even for vials [32], which could increase the cost of experimentation by requiring a greater amount of materials or supplies during the studies [36].

The in vitro aPDT motivated this research on effective and controllable automatic operation time and a variable irradiance light source. The possibility of having a controllable light source for in vitro aPDT in test tubes has notable advantages in comparison with other light sources.

**Figure 10:**
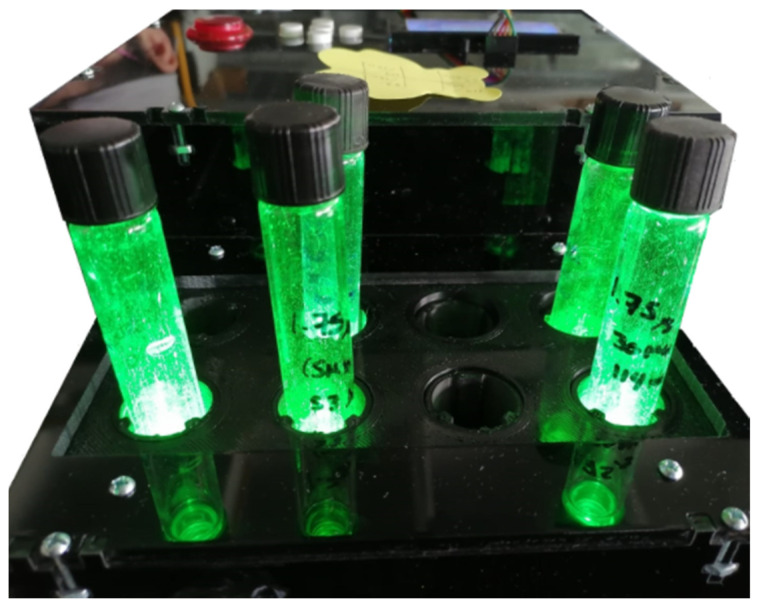
Isolated irradiation for each tube.

For in vitro studies on cell culture plates, recent observations have indicated that non-irradiated wells, on occasion, exhibit similar responses to irradiated ones, particularly when in close proximity (continuous well) [37,38,39]. Nevertheless, this phenomenon was described in the 1970s when elucidating photoirradiation systems for PDT in studies on cancer cells [40]. The effect is applicable to antibacterial inhibition, as uncontrolled irradiation of the sample can lead to complications [41]. The effect of continuous well irradiation in the photodynamic therapy process, known as the bystander effect, is not observed in the developed system due to its configuration because the specially engineered tube rack effectively segregates each sample contained within every tube, as Figure 10 shows.

The overexposure of samples to light can provoke photobleaching of the PS, which could affect the desired reaction or process, hence the importance of having an appropriate dosage in terms of power and irradiation time [42,43]; in addition, the operating temperature of the light source also has an influence [44]. By exerting control over the parameters of the lighting source specifically tailored for this experiment, this effect was effectively mitigated, further underscoring the advantages of the developed system.

Owing to the nature of the PDT, it mainly seeks to maintain a stable temperature in the lighting sources to avoid photothermal effects in the experimental samples. Several systems require the implementation of a temperature control subsystem to avoid it, while other sources only establish methods to maintain a constant temperature using fans or heat sinks; nevertheless, this sometimes limits the operating time [29,30,35].

The green light system allows the irradiation of eight test tubes during adjustable exposure times in intervals of 2.5 min up to one hour. During these operating times, the temperature was monitored and the displacement of the wavelength and the variations concerning the irradiance of the light source unrepresented significant modifications. Despite the temperature rising by 2 °C after 1 h of continuous operation at full intensity using only fans and a heat sink plate in the LED array, to protect the light source due to long periods of operating time, a temperature sensor was implemented in the system.

Some light source systems use a monitoring temperature panel to control the irradiation of the sample and try to fix the temperature and avoid photobleaching and the bystander effect [44]. In this experimental procedure, the temperature sensor registers an increment from 24 °C to 26 °C for all different irradiation power values of the light source. This means that temperature variation was the same as the characterization process. This was an advantage of the developed system to avoid photobleaching of the PS and the bystander effect during the process.

Certainly, in comparison with lighting sources designed for cell culture plates of even up to 96 wells or sources with automatic plate exchange [28,33,35,45], eight tubes seem few, but the implemented source, unlike those mentioned, has control for each LED in terms of operation time and intensity, which allows greater control during experimentation. In addition, the geometry of the tubes allows a test tube rack-type arrangement that isolates the light for each tube, preventing light scattering caused by bacterial cultures and/or colloidal systems.

The individual control of each LED in the system provides the possibility of carrying out experiments with different characteristics, optimizing study times. For example, for the tests with MRSA, a total of nine experiments were carried out, each one with a different concentration. Therefore, different times and irradiances were required, and with fixed irradiation and operation time, it would take 4 h; with the advantage of individual LED control, the experimentation time was reduced to 1 h.

Suzanna Katz et. al. developed an open-source modular Microplate Photoirradiation System for high-throughput photobiology experiments for 24- and 96-well culture cell plates that are controlled using an interface and Wi-Fi communication [46]. Despite having LED control, the difference between the Katz et. al. system and the system presented in this work was that the first needs an interface to control it, and for the green light source used in these experiments, an interface was unnecessary.

On the other hand, the results for the in vitro antimicrobial effect of PDT depend on three factors: endogenic oxygen, an adequate light source, and a PS. A good PS must be (i) nontoxic, (ii) biodegradable, and (iii) available and must have (iv) an appropriate irradiation wavelength and (v) a high production of singlet oxygen [47,48]. In this sense, the synthetic RB dye was selected because it had all the requirements for its use in this study.

RB in combination with green light has been extensively studied with respect to photochemical tissue bonding and corneal transplants [48]. Moreover, the wavelength of the photoactivation light crucial for ROS production must be safe for host (human) cells. Thus, there is no evidence of green light being cytotoxic to the skin, and it may be applied for skin lesion treatment [49].

Photodynamic bacterial eradication involves the utilization of light along with a photosensitizer to trigger a phototoxic response, akin to photodynamic therapy employed for treating skin cancer. Different categories of chemical substances, such as phenothiazines, phthalocyanines, porphyrins, etc., possess photoactive attributes and have been effectively evaluated as agents for photoinactivation against both Gram-positive and Gram-negative bacteria. Photodynamic action relies heavily on singlet oxygen (^1^O_2_), which induces oxidative reactions in the bacterial cell wall, lipid membranes, enzymes, and nucleic acids. The process involves the accumulation of a photosensitizer in or around the cytoplasmic membrane, leading to irreversible damage upon irradiation [50].

“Oxidative stress” is described as an imbalance between the production and consumption of ROS. The oxidative mechanism, if left unregulated, can eradicate microorganisms by causing oxidative harm to proteins, lipids, and genetic material. Bacteria possess defensive proteins against ROS, including enzymes like superoxide dismutase (SOD), catalase (CAT), glutathione peroxidase (GPx), glutathione reductase (GR), the thioredoxin system, and peroxiredoxin (Prxs). When these defense mechanisms fail to neutralize ROS effectively, bacterial cells undergo oxidation due to the heightened oxidative stress [51].

Singlet oxygen is pivotal in initiating oxidative reactions in the vicinity, causing damage to essential bacterial components. This concept forms the basis of the photodynamic inactivation of bacteria [50]. Singlet oxygen has a brief lifespan and moves unrestrictedly within cells. Studies on its propagation indicate that in microorganisms, once generated in the cytosolic membrane’s photosystems, singlet oxygen can potentially spread throughout the entire bacterial cell [52].

Many antimicrobial drugs work by disrupting bacterial functions such as (i) cell wall construction, primarily targeting peptidoglycan, (ii) nucleic acid replication, (iii) protein creation, and (iv) adjustment of membrane permeability. Bacteria possess robust mechanisms for developing or acquiring resistance to these treatments, often regardless of the specific target. Resistance mechanisms are divided into two types, those of intrinsic origin such as the expulsion of the antimicrobial by efflux pumps, neutralization of the antimicrobial by inactivating enzymes, loss of affinity by alteration of the binding site, and restriction of drug entry by alteration of membrane permeability, and extrinsic resistance mechanisms, which are characterized by mutation of existing chromosomal genes, acquisition of foreign genetic material, and mutations of foreign or acquired genetic material [51,53,54].

The reported intrinsic resistance mechanisms of *P. aeruginosa* are alteration of membrane permeability by alteration of the type or amount of porins, efflux pumps of the RND (Resistance Nodulation Division) family, alteration of the site of antimicrobial action as well as activity of aminoglycoside modifying enzymes (AME), and production of Beta lactamases enzymes [55]. In the case of MRSA, intrinsic resistance mechanisms include efflux pumps, enzymatic inactivation by penicillin modifying enzymes (B-lactamases), and alteration of the site of action by penicillin-binding protein 2a (PBP2a) and D-Ala-Dlac [56].

A study conducted by Maisch et al. [50] showed that the production of singlet oxygen relies on the absorption of Photofrin by bacteria, which was examined in two bacterial types. The uptake of Photofrin was quantitatively assessed in *S. aureus* and *E. coli*, chosen as representatives of Gram-positive and Gram-negative bacteria, respectively. *S. aureus* exhibited noticeable Photofrin uptake, whereas no uptake was observed in *E. coli* due to structural differences in their outer cell walls. This observation aligns with previous studies indicating the resistance of Gram-negative bacteria to porphyrin-induced photodynamic effects in the absence of membrane-disrupting agents. The peptidoglycan layer of the *S. aureus* cell wall demonstrates higher permeability, allowing significant Photofrin concentrations to reach the cytoplasmic membrane and remain within the bacteria. Conversely, Gram-negative bacteria possess an outer membrane composed of a lipid bilayer with lipopolysaccharides on the outer leaflet, which restricts Photofrin penetration. In this research, it was found that RB could completely inhibit multidrug-resistant Gram-positive bacteria regardless of the aPDT condition, while the multidrug-resistant Gram-negative bacteria, on the other hand, needed very specific conditions to achieve an inhibition of almost 40%. Although the main factor may be microbial morphology, the sensitivity of bacterial strains also plays a part. As both strains were from clinical origin, microbial inhibition depended not only on morphology but also on the microbial resistance mechanisms they already had. It was worth mentioning that one of the reasons why *P. aeruginosa* did not respond to aPDT compared to MRSA may be because it possesses a virulence factor called pyocyanin, a blue-green pigment, which exerts a protective effect against ROS [57].

It should be noted that for Gram-negative bacteria, conventional aPDT alone is not very efficient due to bacterial morphology, as well as resistance mechanisms exhibited by strains (particularly for *P. aeruginosa*, the main mechanism influencing the efficiency of aPDT is reported to be related to efflux pumps present in the membranes and pores of the bacteria). Therefore, additional and complementary strategies to PDT have been used to increase its antimicrobial effect, such as the use of degrading cell membrane molecules, combination with other therapies (such as Sonodynamic Therapy), and combining PS with antibiotics or peptides [53,58,59,60,61,62].

Nakonieczna et al. [48] provided all light parameters regarding the protocol for the photoin-activation of *P. aeruginosa*, detecting that microbial inactivation improved when light fluence and the RB concentration increased, finding a maximal inhibition rate (>80%) at 60 J/cm^2^ and [RB] = 100 µM. Additionally, experiments with RB in darkness showed that *P. aeruginosa* did not suffer any inhibition caused by the molecule. In this study, it was confirmed that RB concentration and exposure time directly influence the microbial inhibition of *P. aeruginosa*. The results presented in this work show that the greatest inhibition for *P. aeruginosa* is obtained with an irradiance of 17.8 mW/cm^2^ and, therefore, with a light fluence of 32.04 J/cm^2^. This indicates that irradiance and light fluence do not always have to be very high to have an antimicrobial effect.

Data shown in Table 6 confirm the importance of having irradiation equipment and a system that provides adequate conditions, according to the target microorganism, and highlights the effect that the vehicle or the delivery system, where the PS is formulated, may have on microbial inhibition. In this work, the performance of the irradiation equipment was tested with RB in aqueous solutions, but it is expected that its formulation in an appropriate carrier would further improve the inhibition rate, especially for *P. aeruginosa*.

In the present study, a conventional therapy using RB as a PS was employed. However, in recent years, new organic PSs have been identified. These include enhanced natural PSs such as anthraquinones and diacetylcurcumin, as well as synthetic dye derivatives, such as monobrominated neutral red or azure B [68,69,70,71].

However, the use of free PS entails certain restrictions, such as limited biodistribution and bioavailability. To overcome these obstacles, nanotechnology has been used [72,73]. The results have been encouraging, as the ability of PS to penetrate bacterial cells has been increased and self-aggregation of these compounds has been prevented, which is a significant advance [74].

Some nanostructures, such as gold nanoparticles [75,76], carbon nanotubes [66], silica nanoparticles [77], and liposomes [78,79], among others, have been employed in PDT. In addition, fullerenes and quantum dots, which are part of another set of nanostructures, can act as PSs themselves [80,81,82].

In this work, the use of aPDT was restricted to the eradication of bacteria in the planktonic state. However, this method has been investigated as an option to eliminate microbial biofilms [83,84], as well as to disinfect surfaces in hospital settings [85].

## 5. Conclusions

The aim of this manuscript was to present the effect of in vitro aPDT for *Pseudomonas aeruginosa* (*P. aeruginosa*) and *methicillin-resistant Staphylococcus aureus* (MRSA) inhibition using a green light source.

The results of this work showed that using a light source system for test tubes reduces the experimentation time compared with experiments carried out in cell plate culture. An important feature of the equipment presented is that the irradiance is limited to one tube, without affecting neighboring tubes. Working with test tubes allows for a larger culture volume due to the test tube capacity.

Using RB as PS for *P. aeruginosa* and MRSA inhibition through PDT, it was observed that applying light in the correct dosage and adequate RB concentration led to an inhibition rate of *P. aeruginosa* of over 37%. On the other hand, in the case of MRSA, the inhibition was 100% for those irradiated with the green light illumination system presented in this manuscript as a light source for in vitro aPDT in test tubes.

## Figures and Tables

**Table 1:** Factorial experimental design with three factors and one response for both MRSA and *P. aeruginosa* treated with aPDT.

Factor	Units	Low (−1)	High (+1)
RB concentration	µg/mL	1.75	3.00
Exposure time	min	15	30
Green light irradiance	mW/cm^2^	17.8	38.3
Response: Microbial inhibition rate (%)			

**Table 2:** Light source parameters of the green light source system.

Light Parameter	Description and Values
Wavelength (nm)	514 ± 1
Light source technology	LED
Irradiation area (cm^2^)	2.97 ± 0.03
Distance between light and sample (mm)	10 ± 1
Exposure time (s)	900, 1329, 1800
Irradiance (mW/cm^2^)	17.8, 30.3, 38.3
Light fluence (J/cm^2^)	16.03, 32.04, 34.51, 40.86, 69.00

**Table 3:** Results of factorial 2^3^ experimental design with a central point for MRSA aPDT.

Experiment	RB Concentration (µg/mL)	Exposure Time (min)	Green Light Irradiance (mW/cm^2^)	Microbial Inhibition (%)
A	2.38	22.5	30.3	100
B	1.75	30	38.3	100
C	3	30	17.8	100
D	1.75	15	17.8	100
E	3	30	38.3	100
F	1.75	15	38.3	100
G	1.75	30	17.8	100
H	3	15	38.3	100
I	3	15	17.8	100

**Table 4:** Results of factorial 2^3^ experimental design with a central point for *P. aeruginosa*, after treating the samples with aPDT, using RB as PS.

Experiment	RB Concentration (µg/mL)	Exposure Time (min)	Green Light Irradiance (mW/cm^2^)	Microbial Inhibition (%)
A	1.75	15	17.8	2.54 ± 1.16
B	1.75	30	17.8	7.49 ± 0.54
C	3	30	17.8	37.56 ± 0.28
D	3	15	38.3	22.88 ± 0.66
E	1.75	15	38.3	0.71 ± 0.41
F	3	30	38.3	14.17 ± 2.32
G	1.75	30	38.3	5.43 ± 1.02
H	3	15	17.8	6.94 ± 0.45
I	2.375	22.5	30.3	7.98 ± 1.24

**Table 5:** Analysis of variance (ANOVA) to determine factors affecting *P. aeruginosa* microbial inhibition (95% confidence).

Source	Sum of Squares	dF	Mean Squares	F-Reason	*p*-Value
A: RB concentration	1069.13	1	1069.13	25.54	0.0005
B: Exposure time	249.403	1	249.403	5.96	0.0348
C: Green Light irradiance	32.1773	1	32.1773	0.77	0.4012
AB	37.3627	1	37.3627	0.89	0.3671
AC	3.17731	1	3.17731	0.08	0.7886
BC	391.15	1	391.15	9.34	0.0121
Groups	5.87102	1	5.87102	0.14	0.7159
Error total	418.664	10	41.8664		
Total (corr.)	2206.93	17			

**Table 6:** aPDT studies with RB.

Bacteria	Energy Fluence (J/cm^2^)	Exposure Time	RB Concentration	Microbial Inhibition	Irradiation Module	System	Reference
*Escherichia coli* *Serratia marcescens Pseudomonas putida* *Bacillus subtilis*	NE	5 ± 0.1 h 3 ± 0.3 h 4 ± 0.8 h 1.5 ± 0.1 h	0.089 mM	IC_50_ = 22 ± 2 µg/mL IC_50_ = 22 ± 1.7 µg/mL IC_50_ = 29 ± 2 µg/mL IC_50_ = 2.6 ± 1.2 µg/mL	-------	Gelatin nanoparticles	[63]
*Streptococcus mutans*	3.35 6.70 10.05	60 s 120 s 180 s	62 mM	Log 7 reduction	-------	α-cyclodextrin microparticles	[64]
*Pseudomonas aeruginosa*	5.4	15 min	1 mM	89–99% increase in inhibition compared with non-irradiated agar plates	Agar plates in a custom-made plate.	RB solution	[65]
*Escherichia coli*	1674.7	10 min	50 mg/mL	Reduction in the biofilm formation: 64.94 ± 2.91%	-------	Carbon nanotubes	[66]
*Staphylococcus aureus*	18	NE *	0.62 mg/mL	Log 6 reduction	-------	RB solution	[67]

* NE: non specified.

## Data Availability

Data are contained within the article.
